# CKM Gene G *(Ncoi*-) Allele Has a Positive Effect on Maximal Oxygen Uptake in Caucasian Women Practicing Sports Requiring Aerobic and Anaerobic Exercise Metabolism

**DOI:** 10.2478/hukin-2013-0076

**Published:** 2013-12-31

**Authors:** Piotr Gronek, Joanna Holdys, Jakub Kryściak, Daniel Stanisławski

**Affiliations:** 1 Department of Physiology, The Eugeniusz Piasecki University School of Physical Education, Poland.; 2 Computer Laboratory of the Faculty of Animal Breeding and Biology, Poznań University of Life Sciences, Poland.

**Keywords:** CKM gene, athletic performance, genetic polymorphism, metabolic efficiency

## Abstract

The search for genes with a positive influence on physical fitness is a difficult process. Physical fitness is a trait determined by multiple genes, and its genetic basis is then modified by numerous environmental factors. The present study examines the effects of the polymorphism of creatine kinase (CKM) gene on VO2max – a physiological index of aerobic capacity of high heritability. The study sample consisted of 154 men and 85 women, who were students of the University School of Physical Education in Poznań and athletes practicing various sports, including members of the Polish national team. The study revealed a positive effect of a rare G (NcoI−) allele of the CKM gene on maximal oxygen uptake in Caucasian women practicing sports requiring aerobic and anaerobic exercise metabolism. Also a tendency was noted in individuals with NcoI−/− (GG) and NcoI−/+ (GA) genotypes to reach higher VO2max levels.

## Introduction

Creatine N-phosphotranspherase, also known as creatine kinase (CK) or creatine phosphokinase (CPK) plays a crucial role in the homeostasis of cellular ATP by catalyzing the conversion of creatine and consuming adenosine triphosphate (ATP) to create phosphocreatine and adenosine diphosphate (ADP). A high cellular level of creatine kinase with high energy demand, e.g. in striated and non-striated muscles, permits a fast regeneration of ATP supplies, which are the basic source of energy in biochemical processes. CK is found in large volumes in muscle cells. During exercise microleaks and microdamages occur in the myocellular membrane, through which the enzyme enters the bloodstream. Its blood level is indicative of damage of muscle cells – exercise-induced or due to myacardial infarction or ablation (Nigro et al., 1987; Echegaray and Rivera, 2001).

In humans four genes encode subunits of five known types of CK isoenzyme. The muscle type subunit CKM and the brain type subunit CKB form CKMM and CKBB homodimers or a CKMB heterodimer. Their expression is tissue-specific and their activity depends on the demand of individual structures in the cytoplasm: CKMM protein and small amounts of CKMB are found in muscles; CKMB is most active in the heart, while CKBB protein is active in the brain and to some extent in muscle (Fontanet et al., 1991; Wallimann et al., 1992). There are also octameric forms of mitochondrial CK (mt-CK): sarcomeric CK (Scmit-CK) in muscle as well as ubiquitous CK (Umit-CK) expressed in several tissues, e.g. the placenta, retina or sperm. Mitochondrial CK can be found in the intramembrane space of mitochondria, and they are responsible for the replenishment of ATP from phosphocreatine by way of oxidative phosphorylation (Echegaray et al., 2001).

The genes encoding the particular subunits are located on different chromosomes: CKB on 14q32, CKM on 19q13.2-q13.3, Scmit-CK gene on 5q13.3, and Umit-CK on 15q1 (Echegaray et al., 2001). The subject of the present analysis is the CKM subunit gene, which consists of more than 17.5 kbp, 8 exons and 7 introns (Rivera et al., 1997). CKMM protein is specifically linked to the sarcomere M-line – one of heavy meromyosins near myosine ATPase and the external membrane of the sarcoplasmatic reticulum and vesicles. Large amounts of ATP generated near the myosin heads are most likely due to the CKMM activity (Rivera et al., 1997). Several studies have confirmed the hypothesis of the CKM gene being a promising candidate gene affecting the development of aerobic fitness. The muscles of endurance athletes contain more type I slow twitch fibers and feature a high activity of marker enzymes involved in aerobic metabolism. Considering the fact that the activity of CKMM protein in type II fast twitch fibers is twice as high as CKMM activity in slow twitch fibers, a low activity of this enzyme will be another trait of an “endurance” type athlete. This was confirmed by studies of skeletal muscles of knockout mice without the CKM gene. They showed that due to a lower CK activity an improvement of post-exercise endurance parameters can be observed consisting of repeated muscle contractions (Echegaray et al., 2001). It can be assumed that the genetic predisposition to low CKMM protein activity will be advantageous for the development of the endurance phenotype.

So far studies on the CKM gene variants have indicated the presence of multiple polymorphisms, from which two (RFLP) for the NcoI and TaqI endonucleases were examined in view of their association with physical fitness. Both polymorphisms are located near the poly(A) tail at 470 bp and 1119 bp, respectively. The TaqI polymorphism is located in codon 463 and does not cause changes to the amino acid sequence (Rivera et al., 1999).

The present study attempts to analyze the restriction site polymorphism at 1449 of the 3′UTR region of the CKM gene recognized by NcoI, resulting from A>G substitution. A more frequent allele here is the wild-type NcoI+ with a restriction site. In their analysis of association of this polymorphism with an improvement of cardiorespiratory fitness estimated with VO2max after 20 weeks of training, Rivera et al. (1997) noted a smaller change in maximal oxygen uptake in homozygotic individuals with regard to the NcoI-allele in comparison with other genotypes, and thus a worse response to endurance training (Rivera et al., 1997). They also estimated the contribution of the NcoI−/− (GG) genotype to the post-exercise VO2max variability at 9%. On the other hand, Zhou et al. (2006) in their study of volunteers undergoing an 18-week endurance training program noted the greatest training-induced changes of respiratory parameters (inspiratory capacity, resting oxygen consumption) in individuals with the NcoI+/− genotype. Although they did not estimate maximal oxygen uptake, the changes of other spirometric parameters can confirm the contribution of the CKM polymorphism to the development of endurance.

## Material and Methods

### Subjects

The study was carried out on a group of competitive athletes practicing various sport disciplines, representing different sport levels, including representatives of Polish national teams and students of the University School of Physical Education in Poznan, both actively practicing sports, as well as those less active. The study was approved by the Poznan University of Medical Sciences Bioethics Committee, Poland, No 1060/05. Participants were informed about the aim and possible hazards of the analysis and each participant signed a written consent form.

The group of 239 Caucasians (154 men and 85 women) aged 18–26 years was subjected to physiological and genetic analyses. All statistical analyses were performed separately for men and women.

In order to verify the effects of the analysed gene polymorphism on maximal oxygen uptake, depending on the level of physical activity, the participants were then divided into athletes (119 men and 37 women) and non-athletes (35 men and 48 women). Additionally, the athletes were then subdivided into three subgroups classified by the type of exercise metabolism predominating in the discipline they practiced: (i) Sp-St - speed and strength athletes (practicing disciplines with predominance of anaerobic energy metabolism); (ii) E-Sp-St - speed endurance and strength endurance athletes (practicing disciplines requiring both anaerobic and aerobic metabolism); and (iii) E - endurance athletes (practicing disciplines dominated by aerobic energy metabolism). The division of sport disciplines was based on the classification system developed by Bellotti et al. (1978). The Sp-St subgroup contained individuals practicing sprints, long jump, high jump and discuss throw; the E-Sp-St subgroup comprised individuals practising field hockey, tennis, rugby, soccer, volleyball, basketball, handball, boxing, kickboxing, canoeing and rowing; while the E subgroup included triathlonists, medium and long-distance runners, long-distance swimmers, race walkers, skiers and mountaineers.

### Genotyping

Genetic analyses were conducted in the Laboratory of Genetic Analyses at the University School of Physical Education in Poznań, certified by ISO 9001:2008 standards (no. 69178-2009-AQ-POL-RvA). DNA for genetic analyses was isolated from 5 ml of peripheral blood collected from the participants onto anticoagulant (EDTA). DNA isolation was performed using guanidine isothiocyanate (GTC, Sigma) method. An 3′ UTR NcoI CKM polymorphism (1170 bp) was genotyped by PCR-RFLP analysis. DNA was amplified in a volume of 20 μl. Genomic DNA from each examined individual was placed in a separate test tube in the amount of 4 μl (200 ng) and 16 μl reaction mixture was added, containing 50 mM KCl, 10 mM Tris-HCl (pH 8.3), 1.5 mM MgCl2, 0.25 mM dNTP, 7.5 pmol each, primer and 0.5 unit of Taq polymerase (Fermentas Life Sciences, Lithuania). The primers sequence was: Forward - gTg Cgg Tgg ACA CAg CTg CCg and Reverse - CAg CTT ggT CAA AgA CAT TgA gg (Rivera et al., 1999). The 30 cycle reaction was run in a Biometra T-personal thermocycler. The cycle comprised initial denaturation at 95°C for 10 min., denaturation at 95°C – 30s, annealing at 60°C – 30 s, synthesis at 72°C – 30 s and final synthesis at 72°C for 10 min. PCR products were separated on 2% agarose gel. Electrophoresis was run at 100 V for 30 min in Biometra agagel mini horizontal apparatus (Germany) and the results were visualized on a UV transilluminator with ethidium bromide (5mg/ml).

The polymorphism 3′ UTR NcoI CKM gene was genotyped by PCR – RFLP method with NcoI enzyme in the condition recommended by the supplier (Fermentas Life Sciences, Lithuania). The digested products were then electrophoresed in 1.5% agarose gel.

### Maximal oxygen uptake evaluation

In order to determine maximal oxygen uptake the direct method was used during exercise tests on a treadmill (Woodway, USA). During each test, the composition of inhaled and exhaled air (VO2, VCO2) was analysed by an Oxycon Mobile spiroergometer (Jaeger, Germany) and the heart rate (HR) was monitored using a pulsometer (Polar, Finland). The exercise tests were carried out on a treadmill with increasing loads, starting from a running speed of 8 km/h, increasing the load by 2 km/h every 3 min, until the moment of maximum individual load was reached. Physiological analyses were conducted in the Laboratory of Functional Examinations at the University School of Physical Education in Poznań, certified by ISO 9001:2008 standards (no. 69178-2009-AQ-POL-RvA).

### Statistical Analysis

The consistency of the maximal oxygen uptake values and genotype distribution fit to the Hardy–Weinberg principle were verified with the χ^2^ test. The Bartlett test was performed to determine the homogeneity of variance. The association between analysed polymorphisms and maximal oxygen uptake (VO2max) was verified using the ANOVA one-way analysis of variance – the parametric test t. Statistical calculations were performed in the Computer Laboratory of the Faculty of Animal Breeding and Biology at the Poznań University of Life Sciences, with the use of SAS statistical software ver. 9.1 (USA). Statistically significant differences in maximal oxygen uptake between the given genotypes were underlined in tables as a – at p<0.05, A – at p<0.01.

## Results

### Characteristics of subjects

An analysis of association was carried out using results of physiological and genetic studies of 239 subjects. Smokers and subjects outside the age brackets of 18–26 years and normal BMI range were excluded. Individuals for whom there were doubts as to whether their fitness test (treadmill test) results were not maximal due to their low motivation were also excluded from the study protocol. The study sample consisted of 154 men (119 athletes, 37 non-athletes) and 85 women (37 athletes, 48 non-athletes). All subjects were students of the University School of Physical Education, and thus even the non-training controls displayed a higher than average level of physical activity. The subjects trained endurance sports such as the marathon, rowing, kayaking and triathlon; sports involving both aerobic and anaerobic exercise metabolism – field hockey, volleyball, football, handball, rowing; and speed-strength sports such as bodybuilding, track sprints, long jump, and high jump.

### Results of exercise tests

The subjects performed a treadmill test to measure their maximal oxygen uptake levels (VO_2_max) directly with the use of an Oxycon Mobile ergospirometer with constant data transfer from the analyzer to a PC registering changes of such physiological variables as the heart rate (HR), inhaled and exhaled air volume (VO_2_, VCO_2_) and respiratory exchange ratio (RER).

The division of the sample into the subgroups of athletes and non-athletes was justified by the different character of exercise metabolism related to practicing particular sports. The mean VO_2_max values are presented in Tables 1 and 2. As expected the women and non-athletes attained lower VO_2_max levels than men and trained subjects, respectively. Among the athletes the highest maximal oxygen uptake levels were reached by athletes of endurance sports, and the lowest by athletes of speed and strength sports.

**Figure 1:**
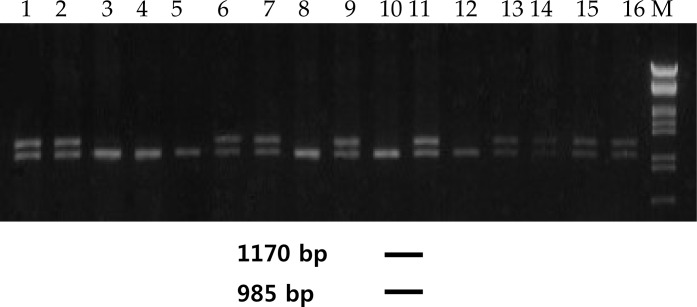
An example of genotyping of restriction site for NcoI within the CKM gene using PCR-RFLP Electrophoresis of products was performed in 1.5% agarose gel. Lanes 1, 2, 6, 7, 9, 11, 13, 14, 15, 16 genotypes of heterozygote NcoI +/−; lanes 3, 4, 5, 8, 10, 12 genotypes of homozygote NcoI+/+; M – size marker λ/EcoRI+HindIII.

The *CKM* polymorphism was genotyped using PCR-RFLP analysis with *NcoI* restrictase and agarose gel electrophoresis. The results are presented in Figure 1

The association of the 3′ UTR *NcoI* polymorphism with the subjects’ VO_2_max levels was examined. In the sample, an analysis of allele and genotype frequency as well as distribution of VO_2_max results was carried out. The χ^2^ test did reveal a normal distribution of the examined variable, and the studied candidate gene was in a genetic equilibrium (χ^2_tab; n−1 = 2, α = 0.05_^ = 5.991; χ^2_tab; n−1=2, α = 0,01_^ = 9.21, χ^2_calc =_^ 3.1930). The homogeneity of variances was checked with the Bartlett’s test. Table 3 demonstrates descriptive statistics and a comparative analysis of VO_2_max levels for polymorphic variants of each studied gene. The analysis of variance did not show statistically significant differences between mean values of the recorded maximal oxygen uptake in groups represented by genotypes GG (*NcoI −/)*, GA (*NcoI −/+)* and AA (*NcoI +/+*).

The analysis of variance did not show statistically significant differences between mean values of the recorded maximal oxygen uptake in groups represented by genotypes GG (*NcoI −/)*, GA (*NcoI −/+)* and AA (*NcoI +/+).*

In order to examine possible differences between the values of maximal oxygen uptake for different genotypes with regard to the level of physical activity the sample was divided into athletes and non-athletes. The distribution of the genotypes and VO_2_max values (minimal, maximal, average) for individual polymorphisms in the two subgroups is shown in Table 4. No statistically significant differences were found.

The analysis of variance did not show statistically significant differences between mean values of maximal oxygen uptake in groups represented by genotypes GG, GA and AA.

The obtained results were also analyzed with regard to VO_2_max levels reached by subjects with different genotypes of the studied polymorphisms in relation to the type of exercise metabolism prevalent in particular sports. Table 5 shows mean VO_2_max values for the polymorphisms of the studied genes in the subgroups practicing speed-strength sports, speed endurance and strength endurance sports, endurance sports and in untrained controls. The analysis of variance revealed in several cases significant differences in VO_2_max, depending on the genotype. There was a significant difference in VO_2_max between female athletes with AA and AG genotypes practicing speed endurance and strength endurance sports, and subjects with AA and GG genotypes (p ≤ 0.01).

The analysis of variance did show statistically significant differences between mean values of the recorded maximal oxygen uptake between either AA and GA or AA and GG genotypes in the female endurance-speed-strength subgroup.

The analysis of variance did show statistically significant differences between mean values of maximal oxygen uptake between either AA and GA or AA and GG genotypes in the female E-Sp-St subgroup.

## Discussion

Athletes feature a considerable inter-individual variability affected by a number of environmental (nutrition, lifestyle, climate, etc) as well as genetic factors (Sawczuk et al., 2013; Karłowska, 2013). Genetic studies aimed at identification of genes responsible for the development of motor traits and physical fitness have been relatively recent (Bouchard et al., 1997). The map of candidate genes that can potentially affect physical fitness becomes larger every year, and currently contains more than 200 genes associated with such aspects as cardiovascular and respiratory fitness; body build and composition - especially muscle mass and strength; carbohydrate and lipid metabolic response to training; and exercise intolerance (Bouchard et al., 1997; Bray et al., 2009).

The inclusion of the genetic component in anatomical, anthropological, biochemical and physiological analyses makes the study of predispositions to practice a given sport more complete. However, an analysis of complex traits being the sums of numerous genes with little individual effect is not easy, and interpretation of results of such an analysis may lead to false conclusions, e.g. due to the small sample size. The perplexity of analysis of association of candidate genes with a complex trait such as physical fitness can be illustrated by ambiguous or even strikingly different study results attained by various research teams examining the genetic profiles of physical abilities in groups of subjects with different levels of physical activity and of different ethnic background.

The present study focused on the effects of a very interesting polymorphism in the CKM gene. Physical fitness was determined by the level of VO2max, which is a good and fairly versatile physiological index of general physical fitness, especially of aerobic capacity. Moreover, maximal oxygen uptake features relatively the highest heritability (h2 = 0.59–0.87) among physical fitness variables, and its value in the age bracket represented by the study sample (18–26 years) reaches its maximum and remains relatively constant (Klissouras, 1971). This is why this particular variable was used for the analysis of association with selected polymorphisms.

To the best of our knowledge the CKM polymorphism has not been studied yet among the Polish population. Our study is arguably the first completed research in Poland aimed at seeking the genetic determinants of physical fitness in Caucasian Polish athletes.

The results of physiological and genetic analysis were checked for conformity with the Hardy-Weinberg equilibrium model. Both VO2max as well as the majority of examined polymorphisms conformed with the Hardy-Weinberg distribution.

So far the RFLP NcoI in the 3′UTR of the CKM gene has been shown to be correlated with VO2max and its changes following 20 weeks of training (Rivera et al., 1997; Rivera et al., 1999). In biochemical tests the post-exercise creatine kinase blood level is indicative of the extent of damage of muscle fibers, i.e. of exercise intensity. The activity of the enzyme is higher in fast-twitch fibers, thus the low activity of creatine kinase will be characteristic in the endurance type athlete. The study confirmed the emergence of a similar mechanism to that of ACTN3, in which the low activity of cellular CKM protein leads to an improvement in aerobic capacity (Rivera et al., 1997). Bouchard et al. (2000) arrived at some surprising results in their search for chromosome regions associated with VO2max and response to training. They revealed a potential linkage between the region of the CKM gene and VO2max changes, contrary to the ACE gene region where the linkage analysis showed no significant influence on maximal oxygen uptake.

The analysis of association between the CKM NcoI polymorphism and maximal oxygen uptake revealed statistically significant differences (p = 0.01) between women with the AA genotype (NcoI+/+) and other genotypes (AG, GG) in a subgroup of athletes practicing sports involving aerobic and anaerobic exercise metabolic patterns. The mean VO2max was, in fact, the lowest for the AA (NcoI+/+) genotype, and the highest for the GG (NcoI−/−) genotype. A tendency to reach the highest VO2max levels by individuals with the GG or AG genotypes can be observed in studies with regard to the sex and genotypes and in the majority of subgroups. It can be concluded that the rare G (NcoI−) allele (as indicated by the low number of individuals with the GG genotype in all subgroups with regard to particular sports) has a positive effect on the VO2max level. The exception are men practicing endurance sports, among whom the GG genotype was the lowest. The observed higher VO2max for the GG (NcoI−/−) genotype does not correspond with other researchers’ findings. Rivera et al. (1997) in their study of baseline VO2max in trained and untrained subjects and VO2max changes caused by endurance training noticed the highest VO2max for the AG genotype and the lowest for the GG genotype. Moreover, the ΔVO2max in response to training was far lower for individuals with the GG genotype, while the examined high responders included no representatives of the GG genotype. Despite the significant association of this polymorphism with maximal oxygen uptake, Rivera et al. (1999) regarded its direct impact on VO2max as rather improbable. They considered, however, its indirect effect on VO2max by way of linkage disequilibrium with another polymorphism of the CKM gene, e.g. TagI, or with genes located nearby, as these genes are related to muscle function (Rivera et al., 1997). Heled et al. (2007) in their analysis of the influence of CKM NcoI on exercise-induced physiological response of the kinase level observed a six times higher CKM blood concentration in individuals with the AA (NcoI +/+) genotype as compared with other genotypes, and associated it with susceptibility to post-exercise rhabdomyolysis. The constant loads applied in that study and the resulting differences in subjects’ post-exercise CKM blood levels indicate that the physiological response was determined by a CKM genotype. They also assumed that the rarer G (NcoI−) gene can serve as a defense mechanism against exercise-induced muscle damage. The effects of the NcoI polymorphism may not be direct, but indirect, through modification of muscle function capacity (Heled et al., 2007). Their study, however, lacked a biochemical background that would allow any further conclusions of the impact on this polymorphism on maximal oxygen uptake.

## Practical implications

An association between the NcoI polymorphism of the CKM gene with maximal oxygen uptake was found in women practicing sports involving both aerobic and anaerobic exercise metabolism. A tendency was observed among individuals with the NcoI−/− (GG) and NcoI−/+ (GA) genotypes to achieve higher VO2max levels.

## Figures and Tables

**Table 1:** *Mean values of maximal oxygen uptake (VO_2_max) in* ml/kg·min^−1^ *in untrained and trained subjects*

**VO_2_max** (ml/kg/min)	**Untrained**					**Trained**				

**Sex**	*N*	*Min*	*Max*	*χ̄*	*SD*	*N*	*Min*	*Max*	*χ̄*	*SD*
**F**	48	30.60	58.40	**42.29**	*5.16*	37	33.80	59.80	**49.91**	*5.85*
**M**	35	42.30	62.20	**50.74**	*4.29*	119	40.30	79.00	**56.42**	*7.21*

F - females, M – males, n – number of participants in the group

Min – the lowest VO2max value in the group

Max - the highest VO2max value in the group

SD – standard deviation

**Table 2:** *Maximal oxygen uptake (VO_2_max) in ml/kg·min^−1^* *in subgroups of subjects with different exercise metabolism profiles*

**VO_2_max**	**Sp-St**					**E-Sp-St**					**E**					**Untrained**				

**Sex**	*N*	*Min*	*Max*	*χ̄*	*SD*	*N*	*Min*	*Max*	*χ̄*	*SD*	*N*	*Min*	*Max*	*χ̄*	*SD*	*N*	*Min*	*Max*	*χ̄*	*SD*
**F**	11	39.30	56.10	**49.45**	*4.65*	9	40.20	52.00	**47.54**	*4.51*	17	33.80	59.80	**51.46**	*6.90*	48	30.60	58.40	**42.29**	*5.16*
**M**	24	41.10	71.50	**54.88**	*5.88*	62	40.30	62.00	**53.84**	*4.24*	33	42.30	79.00	**62.37**	*9.01*	35	42.30	62.20	**50.74**	*4.29*

Sp-St - speed and strength disciplines (disciplines with predominant anaerobic exercise metabolism); E-Sp-S - endurance-speed-strength disciplines (disciplines requiring both anaerobic and aerobic energy resources); E - endurance disciplines (disciplines with predominant aerobic exercise metabolism)

**Table 3:** *Descriptive statistics and comparative analysis of maximal oxygen uptake (VO_2_max) in ml/kg·min^−1^* *between genotypes of the NcoI polymorphism of the CKM gene*

***CKM***	**GG *(NcoI −/−)***					**GA *(NcoI −/+)***					**AA *(NcoI +/+)***				

**Sex**	*N*	*χ̄*	*SD*	*Min*	*Max*	*N*	*χ̄*	*SD*	*Min*	*Max*	*N*	*χ̄*	*SD*	*Min*	*Max*
**F**	6	**47.82**	*8.62*	34.20	58.40	43	**46.75**	*6.64*	31.30	57.70	36	**43.88**	*6.04*	30.60	59.80
**M**	14	**55.07**	*5.39*	49.70	71.50	75	**55.75**	*7.76*	41.10	79.00	65	**54.42**	*6.52*	40.30	72.00

**Table 4:** Descriptive statistics and a comparative analysis of maximal oxygen uptake (VO_2_max) in ml/kg·min^−1^ values between groups of different NcoI polymorphism genotypes of the CKM gene in trained and untrained men and women

**VO_2_max**	***CKM***	**Untrained**					**Trained**				
**Sex**		*N*	*χ̄*	*SD*	*Min*	*Max*	*N*	*χ̄*	*SD*	*Min*	*Max*
**F**	**GG**	3	**44.63**	*12.44*	34.20	58.40	3	**51.00**	*0.79*	50.10	51.60
	**GA**	20	**41.76**	*4.96*	31.30	50.70	23	**51.09**	*4.54*	39.30	57.70
	**AA**	25	**42.44**	*4.33*	30.60	50.50	11	**47.15**	*8.12*	33.80	59.80
**M**	**GG**	2	**51.65**	*2.76*	49.70	53.60	12	**55.64**	*5.58*	50.40	71.50
	**GA**	17	**50.61**	*4.01*	42.30	58.70	58	**57.25**	*7.97*	41.10	79.00
	**AA**	16	**50.76**	*4.88*	42.70	62.20	49	**55.62**	*6.58*	40.30	72.00

**Table 5:** Descriptive statistics and a comparative analysis of maximal oxygen uptake (VO2max) in ml/kg·min^−1^ and different genotypes of NcoI polymorphism in the CKM gene in trained and untrained men and women and subjects practicing various sports, subdivided according to the exercise metabolism profile

	***CKM***	**Sp-St**					**E-Sp-St**					**E**					**Untrained**				
	
**Sex**		*N*	*χ̄*	*SD*	*Min*	*Max*	*N*	*χ̄*	*SD*	*Min*	*Max*	*N*	*χ̄*	*SD*	*Min*	*Max*	*N*	*χ̄*	*SD*	*Min*	*Max*
**F**	**GG**	-					2	**51.4 5A.B**	*0.21*	51.30	51.60	1	**50.10**	**-**	-	-	3	**44.63**	*12.44*	34.20	58.40
	**GA**	8	**50.03**	*5.37*	39.30	56.10	5	**48.70 A**	*2.84*	46.10	52.00	10	**53.14**	*3.94*	46.40	57.70	20	**41.76**	*4.96*	31.30	50.70
	**AA**	3	**47.90**	*1.45*	46.50	49.40	2	**40.75 B**	*0.78*	40.20	41.30	6	**48.90**	*10.51*	33.80	59.80	25	**42.44**	*4.33*	30.60	50.50
**M**	**GG**	4	**56.65**	*9.97*	50.40	71.50	7	**55.11**	*2.52*	52.00	59.50	1	**55.30**	**-**	-	-	2	**51.65**	*2.76*	49.70	53.60
	**GA**	15	**54.15**	*4.74*	41.10	59.90	24	**53.82**	*4.16*	44.30	61.90	19	**64.05**	*9.45*	48.90	79.00	17	**50.61**	*4.01*	42.30	58.70
	**AA**	5	**55.66**	*6.27*	48.40	65.70	31	**53.58**	*4.65*	40.30	62.00	13	**60.46**	*8.36*	42.30	72.00	16	**50.76**	*4.88*	42.70	62.20

*Statistically significant differences in maximal oxygen uptake between the given genotypes was marked by underlining: a – at p* ≤ *0.05, A – at p* ≤ *0.01.*

Sp-St - speed and strength disciplines (disciplines with predominant anaerobic exercise metabolism); E-Sp-S - endurance-speed-strength disciplines (disciplines requiring both anaerobic and aerobic energy resources); E - endurance disciplines (disciplines with predominant aerobic exercise metabolism)
